# Human olfactory dysfunction: causes and consequences

**DOI:** 10.1007/s00441-020-03381-9

**Published:** 2021-01-26

**Authors:** Laura Schäfer, Valentin A. Schriever, Ilona Croy

**Affiliations:** 1 Klinik Und Poliklinik Für Psychotherapie Und Psychosomatik, Medizinische Fakultät Carl Gustav Carus, Technische Universität, Dresden, Germany; 2 Abteilung Neuropädiatrie, Klinik Und Poliklinik Für Kinder- Und Jugendmedizin, Medizinische Fakultät Carl Gustav Carus, Technische Universität, Dresden, Germany

**Keywords:** Olfactory impairment, Short- and long-term consequences, Serious mental health impairment

## Abstract

The sense of smell essentially contributes to social communication, guides nutrition behaviour and elicits avoidance towards environmental hazards. Olfactory smell impairment may hence entail severe consequences for affected individuals. Compared with sensory loss in other modalities, reduced olfactory function is often unnoticed by those affected and diagnosed late. Those patients seeking help frequently suffer from long-term impairments resulting in reduced well-being and quality of life. The current review provides an overview of aetiology, prevalence and specifics of diagnostics in acquired and congenital olfactory loss and focusses on short- and long-term consequences. Compensation strategies are elaborated, and treatment options are mentioned. Individual characteristics associated with the development of serious mental health impairment are discussed in order to help practitioners identifying populations at risk.

## Causes of olfactory impairment and prevalence in human

The smell of freshly baked bread, salty sea air or strawberries in summer are all sensory impressions enriching our lives. Yet, an intact sense of smell is often not worth mentioning until symptoms of olfactory loss are experienced. Although mainly unconscious for the perceiver, olfaction guides human behaviour in various life domains—nutrition, avoidance of harm, sexual interaction or social communication (Stevenson 2010). Impairments in daily life often become apparent even before the olfactory disorder is diagnosed. About one third of patients seeking professional help reports significant diminished life quality (Croy, Nordin, and Hummel 2014), and as olfactory disorders affect 3% up to 22% in the general population, early detection and treatment strategies are essential to provide (for a review, see Boesveldt et al. 2017; Desiato et al. 2020; Hoffman, Rawal, Li, and Duffy 2016).

Olfactory disorders can be divided into quantitative and qualitative. Within the group of quantitative disorders, the variety of reported prevalence for a complete loss of the olfactory function (functional anosmia) ranges from of 3.6 to 5.8% within the general population (for a review, see Hummel et al. (2017). In contrast, a specific anosmia, the inability to smell one certain odour while other odours are perceived normally, is normal and a non-pathological phenomenon (Croy et al. 2015; Zou et al. 2020). About 13–18% of the population are affected by partial impairment of quantitative function (hyposmia; Hummel et al. 2017), while qualitative impairment (e.g., parosmia) occurs with an estimated prevalence of 3.9% (Nordin, Brämerson, Millqvist, and Bende 2007). As mentioned above, the reported prevalence of olfactory dysfunction ranges from 3 to 22%. This difference is due to the demographic characteristic of the study population. Olfactory dysfunction increases with age (Doty, Shaman, Applebaum et al. 1984a, b) and even predicts mortality within old individuals (Pinto, Wroblewski, Kern, Schumm, and McClintock 2014), while the prevalence is assumed to be low in a paediatric population (Oozeer, Forbes, Clement, and Kubba 2011).

Anosmia does not imply that all chemosensory function is lost; most patients are still able to perceive trigeminal sensations (J Frasnelli, Schuster, and Hummel 2007). Quantitative olfactory dysfunction can either be acquired after birth or congenital. The latter one includes genetic conditions and prenatally acquired diseases with associated olfactory dysfunction.

Acquired olfactory dysfunction: Attempts have been made to classify the aetiologies of acquired olfactory dysfunction according to the location of the presumed pathology. Three categories have been identified: (1) conductive dysfunction, (2) sensorineural dysfunction, and (3) central dysfunction (Hummel et al. 2017; Walker, Pottinger, Scott, and Hopkins 2020). Not all aetiologies of acquired olfactory dysfunction can be easily assigned to one of the three categories, and an overlap in mechanisms resulting in olfactory dysfunction can be observed. Most frequently, olfactory dysfunction is acquired due to sinonasal disease (30%), upper respiratory tract infection (URTI, 25%), traumatic brain injury (TBI, 14%) or unknown (idiopathic, 12%) (Keller and Malaspina 2013). A current example of post-viral olfactory loss is olfactory impairment in covid-19 patients (Parma et al. 2020). Besides, neurodegenerative disorders or toxins can lead to olfactory dysfunction (Hummel et al. 2017). The frequencies of aetiologies of olfactory dysfunction change with age (Johannes Frasnelli, Fark, Lehmann, Gerber, and Hummel 2013; Keller and Malaspina 2013), while the frequency of olfactory dysfunction due to TBI decreases with age, the frequency of other aetiologies such as URTI or idiopathic increase with age (Schriever and Hummel 2020). In paediatric populations, particular attention should be paid to adenoid hypertrophy, autism or traumatic brain injury as children suffering from those disorders exhibit an elevated risk of accompanying olfactory impairment (for a detailed review, see Valentin A Schriever, Gellrich, von der Hagen, and Hummel (2018).

Congenital olfactory dysfunction: It has been estimated that 0.01–0.02% of the general population are born without a functioning sense of smell (Croy, Negoias, Novakova, Landis, and Hummel 2012). Whereas genetic variations account for a minor part of congenital anosmia (CA), i.e., in the Kallmann syndrome, which is characterized by olfactory dysfunction (anosmia or hyposmia) and isolated hypogonadotrophic hypogonadism (IHH), the majority of CA patients lacks known genetic variations or underlying diseases. This phenomenon is then called an *isolated* congenital anosmia (ICA, (Karstensen and Tommerup 2012)) and is supposed to be genetically or infectiously caused or to be initiated by stress events occurring in pregnancy or soon after birth (Abolmaali, Hietschold, Vogl, Hüttenbrink, and Hummel 2002). Although the aetiology is not yet completely understood, parallel occurring morphological neuropathological alterations have been evident. Those include reduced depth of the olfactory sulci (which in turn can serve as a proxy for the presence of olfactory tracts), as well as hypo- or aplastic olfactory bulbs (Abolmaali et al. 2002; Yousem, Geckle, Bilker, McKeown, and Doty 1996). Compared with other congenital sensory impairments, which are screened on a regular base in paediatric routine care, ICA is detected late (Bojanowski, Hummel, and Croy 2013). On average, patients (or their parents) observe first signs around the age of 10 years, and another 13 years remain until final diagnosis (Bojanowski et al. 2013). Another characteristic of ICA is that the congenital absence disturbs development less severely than blindness or deafness, but still affects everyday situations, such as detection of spoiled food or a strong body odour (Bojanowski et al. 2013). In order to diagnose ICA, a multidimensional clinical assessment is obtained comprising all of the above-mentioned steps. The olfactory assessment should be based on appropriate diagnostic tools including normative data for children and adolescents (Gellrich et al. 2019; Valentin A Schriever, Agosin, et al. 2018a, b). As morphological brain changes are a valid indicator for ICA, structural imaging should also be included (Bojanowski et al. 2013). Imaging studies have demonstrated that acquired anosmia is associated with decreased grey matter volume in secondary olfactory structures (e.g., nucleus accumbens, medial prefontral cortex; Bitter et al. 2010), which does not apply for congenital dysfunction. Corresponding to findings from other sensory modalities, e.g., congenital blindness, previous studies found associations between ICA and enhanced brain volume as well as thickness in primary (piriform and entorhinal cortex) but also secondary (orbitofrontal cortex) olfactory structures (Johannes Frasnelli et al. 2013). It has been suggested that this is a consequence of diminished peripheral sensory input which impedes the process of synaptic pruning during brain development (Johannes Frasnelli et al. 2013). A recent published study, however, revealed a slightly different picture: Peter et al. (2020) observed that morphology of ICA individuals did not differ from healthy controls in primary structures, namely piriform cortex, but an altered brain structure was observed in secondary areas, namely atrophied olfactory sulci, as well as enhanced thickness and grey matter volume in medial orbital gyri.

## Delayed diagnostic procedure

Introducing the following paragraph, we can imagine for a moment that our eyesight is rapidly deteriorating. Most people would quickly consult a doctor in such a case. Olfactory loss is a different matter (for a review, see Boesveldt et al. (2017)). A majority of affected patients seems not to be aware of their impairment, even when directly queried (Adams et al. 2017). Asking people aged 50–100 years about changes in their sensory functions, they indicate losses in seeing and hearing, but do not state changes in the sense of smell. Moreover, subjective ratings corresponded with objective assessment for vision and audition, but diverged for the sense of smell (Cavazzana et al. 2018). Not only older people tend to overestimate their olfactory abilities: In a study investigating 9139 people who reported to have normal olfaction, functional anosmia was detected in 3.4% (Oleszkiewicz and Hummel 2019). Although the prevalence was highest in the older age group, a considerable number of middle-aged people were found to be functionally anosmic. Sample characteristics of the functional anosmia group revealed that a higher proportion of women compared with men were affected and the affected women were older. Similarly, A Oleszkiewicz, Kunkel, Larsson, and Hummel (2020) found one out of three individuals to underestimate their olfactory abilities, which is not surprising, as the sense of smell is often little appreciated (Boesveldt et al. 2017). The poor sensitivity of self-reported olfactory impairment suggests that for the majority of those affected, the individual suffering is not sufficient to seek treatment (Oleszkiewicz et al. 2020).

The lack of ability to subjectively assess olfactory performance may hinder an early diagnosis. As a consequence, the unnoticed impairment may cause exacerbation of side effects. Although a majority of anosmic individuals does not appear to be bothered in daily life, a considerable number of patients declare negative effects of their sensory loss, often manifested in reduced life quality or depressive symptoms (Croy et al. 2014). From that point of view, the development of low-threshold screening tools could hence promote early detection of smell disorders before adverse long-term effects arise. In the case of self-report, Hoffman et al. (2016) showed, i.e., that adding specific questions to the anamnestic procedure (e.g., on age-related olfactory changes) can increase the screening sensitivity. If olfactory dysfunction is already suspected (e.g. in case of a posttraumatic olfactory loss), a detailed anamnesis questionnaire should be provided as standard, as well as otolaryngologic physical examination (for a detailed description, see Hummel et al. 2017; Costanzo and Miwa 2006). Besides, comprehensive olfactory assessment requires objective measurement, e.g., the derivation of event-related potentials (ERPs) in reaction to odourant stimuli emitted by an olfactometer (Güdücü et al. 2019) or standardized psychophysiological tests in which the patients have to complete tasks on identification, detection and discrimination of the odour. State of the art tools are, e.g., the Sniffin’ Sticks (T Hummel, Kobal, Gudziol, and Mackay-Sim 2007) or UPSIT procedure (Doty, Shaman, Kimmelman, and Dann 1984a, b) presenting the patient to odours contained in pen-like sticks or test strips. Such sensory assessment can be easily implemented in routine care settings serving as an efficient diagnostic tool in order to provide early detection of smell impairment.

## Potential consequences of olfactory impairment

**Fig. 1:**
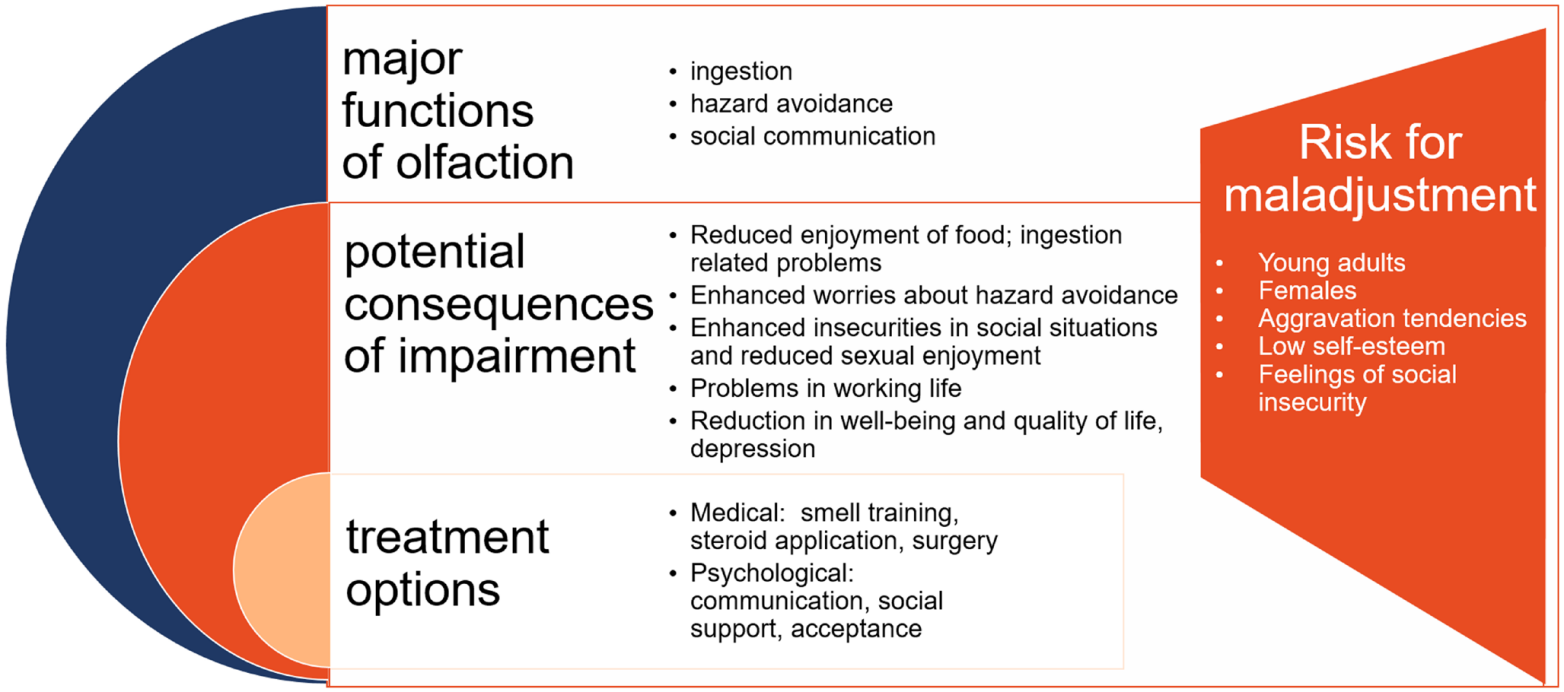
Consequences of olfactory dysfunction: Overview of the functions of smell, consequences of smell impairment and treatment approaches which specifically should consider populations at risk for individual maladjustment

Independent from its cause, patients report similar impairments when it comes to the impact of anosmia on daily life functioning. Areas initially disturbed by the olfactory loss include safety, hygiene, work-related issues or social interaction (Croy et al. 2014). This relates well to the major functions of olfaction, which Stevenson (2010) named (1) ingestion, (2) hazard avoidance and (3) social communication. In the following, the implications of olfactory dysfunction are outlined separately for each cluster (compare Fig. 1).

### Ingestion

Detection of edible food, rejection of spoiled food regulation of appetite and hedonic experience such as flavour perception—all those features of nutrition are mediated by chemosensation (Stevenson 2010) and hence affected in anosmia.

The altered perception of food is often an initial sign of olfactory loss which is noticed by the patients themselves. It frequently leads to dysregulated appetite and hence altered food intake (Aschenbrenner et al. 2008; Mattes et al. 1990) but also affects food preparation, in terms of cooking or the recognition of rotten food (for a review, see Stevenson (2010)). ICA patients report mainly problems with the detection of spoiled food but do not differ in eating behaviour when compared with normosmic individuals (Croy et al. 2012). In contrast, patients with acquired olfactory dysfunction often report altered eating behaviour, as well as anhedonia in relation to food. Up to 70% of patients with acquired anosmia report a decreased pleasure of eating or drinking (for a review, see Croy et al. (2014). This is reflected by decreased perceptions of pleasantness, intensity and appetite (such as reduced liking of chocolate) in comparison with normosmic individuals, and those findings cannot be explained by differences in salivary flow (Zang et al. 2019). With regard to the aetiology of olfactory loss, specific correlates of eating-related impairment have been observed: I.e., in chronic rhinosinusitis, 23% of the patients complain about reduced quality of life associated with eating. The degree of this reduction was predicted by aspirin-exacerbated respiratory disease, as well as by depressive symptoms (Rowan et al. 2019). An increased or decreased amount of food intake over a long period of time (e.g., Keller and Malaspina (2013) can, i.e., lead to obesity and thus elevate the risk not only for eating disorders (Fairburn et al. 1998) but also for subsequent cardiovascular, endocrinological or metabolic diseases (Van Gaal, Mertens, and Christophe 2006). Indeed, investigations of patients with type 2 diabetes exhibited repeatedly lower olfactory function (for a review, see Hillson (2014), as well as gustatory impairment, especially in complicated cases. Although these observations are correlational, they reflect a close interdependence of chemosensation with diabetes and related eating behaviour. For example, difficulties in sensing sucrose and glucose are assumed to be responsible for elevated sugar intake and resulting hyperglycemia (Hillson 2014).

Further consequences of obesity may relate to a reduced quality of life (Dieterle and Landgraf 2006) and negative psychosocial consequences, e.g., the experience of discrimination (Myers and Rosen 1999; Star, Hay, Quirk, and Mond 2015), which affects self-esteem and in turn is a risk factor for developing further psychological impairment, such as depressive symptoms (Orth and Robins 2013).

### Hazard avoidance

Odours serve as warning signals, e.g., for the detection of gas or hygiene-related domains. Related to this, body odours can signal disease status (Olsson et al. 2014) and thus elicit avoidance reactions and feelings of disgust. In addition, associations between a certain odourant and a dangerous situation are easily learned (Van den Bergh et al. 1999).

Those signalling functions of odours are disturbed in anosmia. Hence, difficulties in detecting danger (e.g., by gas leaks) or disease might lead to infection or staying in situations at risk, and indeed, ICA patients reported more frequent accidents in household compared with healthy controls (Croy et al. 2012). Besides the actual danger of those situations, the major concerns of anosmic patients are feelings of insecurity and worrying about safety issues due to their deficits (Blomqvist, Brämerson, Stjärne, and Nordin 2004; Croy et al. 2014; Nordin et al. 2011). Patients with olfactory loss further state to be bothered by hygiene-related impairment. This is reflected by the patient’s inability to detect one’s own body odour and relating insecurity, e.g., the fear of smelling bad or having difficulties in perfume usage, as well as the inability and resulting shame when it comes to detect other’s body odours, e.g., when a child’s diaper needs to be changed (Blomqvist et al. 2004; Croy et al. 2014; Keller and Malaspina 2013; Nordin et al. 2011; Temmel et al. 2002). Although ICA patients do not exhibit altered behaviour compared with healthy controls when it comes to personal hygiene, e.g., frequency of showering or washing clothes; (Croy et al. 2012), worries on that domain may affect well-being on the long run and lead to serious consequences for mental health. Extended worrying, which is characterized by “anxious affect during repetitive thought about possible future threats,” (Borkovec, Robinson, Pruzinsky, and DePree 1983; McLaughlin, Borkovec, and Sibrava 2007) relates to negative affect and can exacerbate to the development of depressive or anxiety symptoms (McLaughlin et al. 2007). In this context it is noteworthy that questionnaires asking for hazards in relation to olfactory dysfunction are often aimed at assessing fears but not at actual undetected fires or gas leaks, presumably due to their low incidence.

### Social communication

The sense of smell is remarkably linked to the initial experience of human bond, the tie between mother and child (Porter 1998; Porter, Cernoch, and McLaughlin 1983). Olfaction plays a key role in a newborn’s orientation towards the environment. The human olfactory system is developed in utero, and human neonates exhibit transient olfactory abilities between pre- and postpartum period (Schaal, Marlier, and Soussignan 1998; Schaal, Saxton, Loos, Soussignan, and Durand 2020). The newborn’s behaviour is strongly guided by means of olfaction, i.e., the ability to detect maternal odour a few hours postpartum or crawling towards the mother’s breast (Varendi and Porter 2001), which presumably contributes to breastfeeding. To our knowledge, there is only one study up to date exploring the effect of congenital anosmia on breastfeeding and this study did not observe differences in breastfeeding between ICA patients compared with controls (Croy et al. 2012). ICA individuals did however report to have received less maternal care than controls (Novakova, Croy, Havlicek, and Hummel 2011). This result should be interpreted carefully as it may have been biased by highly selective sampling and retrospective interviewing, which is prone to memory bias.

Research on chemosignals has revealed that social situations are strongly informed by body odours. They do not only signal emotional state, such as anxiety (Prehn-Kristensen et al. 2009), but also guide attribution of personality characteristics—a negatively perceived body odour is associated with unappealing traits, e.g., being “unfriendly” (McBurney, Levine, and Cavanaugh 1976). Evidence from imaging studies shows neural activation in social processing areas in response to body odours suggesting that those are relevant for interpretation of social situations (Lübke et al. 2014; Lundström, Boyle, Zatorre, and Jones‐Gotman 2009; Schäfer, Hummel, and Croy 2019a, b). The strength of this connection seems to be related to the degree of social openness (Lübke et al. 2014).

In particular, olfaction contributes to intimate relationships—body odours signal kinship (Porter 1998), transport information on genetic similarity (Milinski, Croy, Hummel, and Boehm 2013) or developmental status. This is relevant within the mother–child relationship (Schäfer, Sorokowska, Sauter, Schmidt, and Croy 2020a, b; Schäfer, Sorokowska, Weidner, and Croy 2020a, b). Bonding difficulties are associated with the inability to recognize the own baby’s odour, and mothers with impaired bonding do not perceived their own child’s odour as pleasant as healthy mothers (Croy, Mohr, Weidner, Hummel, and Junge-Hoffmeister (2019)). Mate choice and romantic relations are also influenced by olfactory cues (for a review, see (Mahmut and Croy 2019), as they impact attractiveness perception, presumably dependent on genetic (e.g., Sorokowska et al. (2018) or for review (Havlíček, Winternitz, and Roberts 2020)) or hormonal traits (Lobmaier, Fischbacher, Wirthmüller, and Knoch 2018). With regard to sexuality, olfaction contributes to quality and quantity of sexual behaviour (Bendas, Hummel, and Croy 2018) and the experience of sexual arousal is mediated by body odour perception (Cerda-Molina, Hernández-López, Claudio, Chavira-Ramírez, and Mondragón-Ceballos 2013).

About one third of the patients with acquired olfactory dysfunction report a negative impact of their disorder on a social domain (Croy et al. 2014), especially when it comes to romantic relationships (for a review, see (Mahmut and Croy 2019)). Individuals suffering from acquired olfactory loss name to be affected in sexual experience (i.e., missing to smell their partner’s body odour) as well as in sexual desire (Schäfer, Mehler, et al. 2019a, b).

Likewise, congenital olfactory loss has been demonstrated to affect both, quantity and quality of sexual behaviour. While men with ICA report a diminished number of sexual relationships, women state an increased insecurity about partnership due to their impairment (Croy et al. 2013). In addition, they exhibit greater feelings of social insecurity compared with healthy controls (Croy et al. 2012).

In this respect, long-term consequences have to be considered, as feelings of insecurity and social anxiety are linked to lower self-esteem or social isolation (De Jong, Sportel, De Hullu, and Nauta 2012; Lim, Rodebaugh, Zyphur, and Gleeson 2016). Here, a special awareness should be raised towards young women, because women generally report to rely more on olfactory cues than men (Havlicek et al. 2008; Murr, Hummel, Ritschel, and Croy 2018) and because impeded olfactory function is assumed to be particularly crucial in young adult age, as this period is associated with formatting bonds towards mates or towards the own baby.

In sum, these findings highlight the importance of olfaction for social communication. Yet, they demonstrate only correlative associations and no causal statements can be derived. Furthermore, this findings rely on questionnaires and it is unclear how actual behaviour, e.g., when it comes to dating (Mahmut and Croy 2019), differs between hyposmic and normosmic people. Further experimental studies are necessary in order to elucidate the observable behavioural impact of anosmia.

### General quality of life and anhedonia

Although the majority of patients deal well with olfactory impairment in their daily life, about one third of those individuals with olfactory loss who seek professional help states substantial reduction in overall life quality (Bojanowski et al. 2013). Individual reports thereby exhibit a wide range of areas (e.g., memory, family relationships, celebrations; Erskine and Philpott 2020) in which the loss of sense comes along with anhedonia and related negative feelings, such as frustration, isolation, anxiety or sadness (Bojanowski et al. 2013; Erskine and Philpott 2020) the inability to smell flowers, perfume or the beloved partner causes individual suffering and lowers general well-being. A patient’s quote (who was interviewed in a study of Keller and Malaspina (2013) illustrates this impressively: “The sad thing, I find, is not being able to appreciate the everyday smells which we take for granted: perfume, freshly mown grass, freshly baked bread, scent of bluebells/roses/flowers in general. Living by the sea, I used to love the smell of the seaweed around the tide pools. The list is endless.“

Based on shared central neural processing pathways between olfaction and emotion generation (e.g., Gottfried 2006; Rochet 2018), the overlap between olfactory dysfunction and anhedonia is not puzzling. Depressive symptoms are higher than expected by chance in patients with acquired, as well as in patients with congenital anosmia (for reviews compare, e.g., Croy and Hummel 2017; Kohli, Soler, Nguyen, Muus, and Schlosser 2016; Schablitzky and Pause 2014). Different pathways may contribute to this phenomenon: Altered olfactory bulb functioning may lead to diminished neural input and hence imbalanced neurotransmission in limbic and reward brain circuits which may cause depressive symptoms, or depression may lead to withdrawal behaviour which results in reduced sensory input and hence decreased olfactory stimulation and performance (Croy and Hummel 2017). Evidence for the first explanation comes from a study showing that hyposmic individuals have reduced processing and perception of visual emotional stimuli (Han, Hummel, Raue, and Croy 2019). Apart from these neurological pathways, the experience of olfactory loss itself and related impairments can of course lead to reduced quality of life up to depressive symptoms and this development is even more likely in people with low self-esteem (Kollndorfer, Reichert, Brückler, Hinterleitner, and Schöpf (2017)). This link should hence be considered when explaining individual vulnerability and emergence of depression in olfactory dysfunction (Orth and Robins 2013).

In sum, closer examination of the here discussed life domains revealed long-term costs associated with olfactory loss. Recent research however indicates that emphasis on negative consequences of olfactory deficits should be done with caution. A Oleszkiewicz et al. (2020) assessed effects of undetected olfactory impairment in 203 individuals all claiming normal smell abilities. Strikingly, individuals affected by olfactory loss did not differ from healthy individuals in terms of emotional functioning, mental and general health. Lower performance of subjects with olfactory impairment was found for subdomains of cognition and physical functioning. In addition, they reported weaker associations between odours, emotions and memories, which might be relevant, i.e., in linking body odours to romantic feelings. However, no differences in overall social recognition accuracy were observed. The study illustrates that only few life domains are affected by olfactory loss in individuals not seeking treatment.

Yet, patients being aware of their deficits should receive special attention when discussing long-term costs of olfactory loss. Although patients with quantitative olfactory disorders seem to be less bothered than patients suffering from qualitative olfactory disorders and less disturbed than hyposmic patients (as those are able to sense olfactory percepts and are hence more often confronted with their inability (Frasnelli and Hummel 2005), negative effects on the long run are reported for a substantial number of patients seeking professional help (Blomqvist et al. 2004; Croy et al. 2014) and thus should be taken into account when it comes to clinical practice.

In general, women report greater disturbance of olfactory impairment than men (Frasnelli and Hummel 2005), especially when it comes to affected social and domestic life (Philpott and Boak 2014); however, a recently published meta-analysis reveals men to be more affected by olfactory loss than women (Desiato et al. 2020). In that regard, a look at the individual importance of olfaction is helpful in order to capture relevant dimensions of potential damage due to olfactory dysfunction. It further serves identifying populations at risk and allows for addressing these patients systematically, e.g., in offering a targeted screening for concomitant psychological disorders. A recent questionnaire study exploring the individual importance of olfaction among a wide sample of normosmic and dysosmic individuals aged 15–82 years asked for application (e.g., smelling clothes in order to check for washing), association (e.g., emotional associations with certain smells) and consequences (e.g., decision making based on olfactory cues) of olfaction (Murr et al. 2018). Within the normosmic group, young women exhibited the highest individual significance of their sense of smell. The data showed also that impaired olfaction was linked to a lower individual attribution of its significance. Additionally, about one-fifth of dysosmic individuals tended to aggravate their symptoms, which hints at maladaptive coping with the disorder and associated distress. These findings suggest that a particular sensitivity should be raised towards (a) young women and (b) affected patients showing aggravating tendencies when assessing potential negative side effects of anosmia.

In all cases, a comprehensive analysis of individual conditions should be carried out, as those can increase as well as attenuate the occurrence of negative outcomes and mediate well-being. This involves conditions on a physiological, psychological and interpersonal levels. On a physiological level, it is important to distinguish specific features of olfaction. While olfactory performance in most studies is measured by orthonasal functioning (e.g., by Sniffin’ Sticks assessment), retronasal qualities of olfaction should not be neglected (Anna Oleszkiewicz et al. 2019). These are involved in flavour processing thereby accounting for hedonic perception of food and impairment on that domain is a frequent initial complain of patients with olfactory loss. Oleszkiewicz et al. (2019) tested indicators of life quality in patients with olfactory impairment and observed that taste identification contributed stronger to health outcomes than orthonasal olfaction, and moreover, was the only significant variable predicting quality of life. On a psychological level, well-being in patients with olfactory loss was positively predicted by the willingness to communicate, and this effect was enhanced in individuals reporting greater support by their spouse (Hofsöe, Lehane, Wittich, Hilpert, and Dammeyer 2019). Hence, communication and support seem to facilitate coping with the impairment.

## Compensation

Patients with olfactory dysfunction use various strategies in order to cope with their deficits, from installing a gas detector to the devaluation of the personal meaningfulness of their sense of smell (Croy et al. 2014). As a frequent strategy focused on problem solution, smell disorder patients state to involve other people (e.g., family members)—either in order to test food which might be rotten or to check the patient’s perfume usage (Blomqvist et al. 2004; Nordin et al. 2011). The most widely used emotional adaption strategy, namely “acceptance of the situation and making the best of it,” is reported to be used by about three-fourth within different samples of smell disorder patients. Further strategies targeting emotional adaptation include “seeking social support” or “comparing problems with people who are worse off” (Blomqvist et al. 2004; Nordin et al. 2011). In the following, different aspects of compensation within each major function of olfaction will be elucidated.

### Ingestion

In order to compensate for altered sensation when it comes to eating, patients cope with increased usage of salt or other spices in order to intensify the taste (Keller and Malaspina 2013). Additionally, they apply schedules for eating in order to deal with dysregulated appetite (Croy et al. 2014). Although rejection of spoiled food is still possible as there are other senses involved, ICA patients state widely problems in that regard (Croy et al. 2012), and therefore frequently ask family members for help (Blomqvist et al. 2004).

### Hazard avoidance

Evidence on compensatory hygiene-related behaviour is mixed. While Miwa et al. (2001) reported that patients with olfactory deficits are more concerned about cleaning of their house, stated to wash clothes more frequently and use deodorants to a greater extent when compared with a control group, Croy et al. (2012) did not observe such differences in personal hygiene-related behaviour. A reason for that discrepancy might be that both studies used different questionnaires and that Miwa et al. (2001) investigated a heterogenous sample of patients with olfactory impairment, while Croy et al. (2012) focused on ICA patients only. It thus can be assumed that ICA patients develop early strategies in life to cope with those issues.

However, ICA patients do still report a greater risk of household accidents when compared with healthy controls (Bojanowski et al. 2013; Croy et al. 2012).

### Social communication

From other modalities it has been evident that the loss of one sense can lead to compensatory amplification in another domain, i.e., increased auditory abilities in case of blindness (for a review, see (Kupers and Ptito 2014). Similar mechanisms are supposed to apply for olfaction, but to our knowledge, only one study has directly tested this assumption so far using socially relevant stimuli: Lemogne et al. (2015) assessed differences in visual emotion recognition between anosmic patients and healthy controls. Therefore, participants were exposed to facial stimuli and had to detect changes in emotional expression. Results showed specific patterns depending on the emotion displayed and on the aetiology of olfactory loss: Patients suffering from congenital, but not from acquired anosmia, exhibited lower error rates than healthy controls for the detection of fearful and disgusting facial expression. Specific analyses of the acquired anosmia group revealed a negative association between duration of olfactory loss and performance—less errors were reported for patients with longer duration of the disorder. In sum, the study provides first evidence regarding compensatory sensory mechanisms in congenital anosmia.

## Treatment options

Several interventions exist for treatment of olfactory disorders, which differ depending on aetiology. They include smell training, steroid application or surgery (for a detailed overview, see (Boesveldt et al. 2017)). While some approaches have been shown to promote partial restorage of the olfactory abilities, the development of successful interventions is still in its infancy.

Complementary, gaining emotional acceptance of the deficits is crucial in order to enable adaptive adjustment to the disease. The majority of affected patients is well able to adapt to their loss by means of lowering the individual importance of olfaction in daily life activities (Croy et al. 2011). Such a mechanism of detachment to the sense is assumed to sustain well-being notwithstanding the experience of significant deficits (Modinos, Ormel and Aleman 2010).

From other modalities it is known that adjustment to sensory loss (e.g., blindness or deafness) is a continuous, dynamic process involving adaptive coping strategies, such as active coping, namely focusing on positive aspects, as well as “realistic emotional acceptance” of the situation (Fitzgerald and Parkes 1998; Lehane 2017; Olze et al. 2011). Those coping styles relate to enhanced self-esteem and quality of life. In turn, maladaptive coping, such as evasion or distraction is associated with negative outcomes, being reflected in reduced life quality or depression (Fitzgerald and Parkes 1998; Lehane 2017; Olze et al. 2011).

In case of negative concomitant effects of the olfactory loss, analysis of the respective coping behaviour should be carried out first in order to detect maladaptive strategies, which may disturb the adjustment process. Based on this, adaptive coping strategies can be developed considering individual resources of the patient. Such strategies comprise open communication and active seeking for social support as well as practicing mindfulness, which includes acting, observing or describing (of emotions or thoughts) leading to acceptance without judgement (Kabat‐Zinn 2003; Prazak et al. 2012). Mindfulness promotes well-being on psychological and physiological outcomes (Prazak et al. 2012). In that regard, reappraisal, the capacity of detachment to negative affect and the openness to do so, has been shown to successfully reduce adverse emotions on a neural level (Modinos, Ormel, and Aleman 2010). In turn, such abilities may help to achieve adaptive adjustment to olfactory deficits by favouring positive outcomes in terms of well-being and self-esteem.

## Conclusion

Although a vast majority of patients with olfactory dysfunction seems to deal well with the olfactory loss, some suffer from concomitant impairment on several life domains. This can result in reduced well-being, the development of depressive symptoms and overall reduced quality of life. Delayed diagnostic procedure, e.g., due to poor sensitivity of self-report, may hinder prompt adequate treatment. When identifying populations at risk, particular attention should be paid to patients exhibiting aggravating tendencies as well as to young women as those report a high individual significance of olfaction in their daily life. Patients with olfactory dysfunction dispose a wide array of compensation strategies which comprise emotion- and problem-focused adjustment to the disorder. In addition, promising treatment options are available aiming at partial restorage of the olfactory function. At the same time, strategies that convey acceptance of the disorder should be promoted, as this reflects a key feature of adaptive coping and is linked to improved mental health outcomes.

## References

[CR1] Abolmaali ND, Hietschold V, Vogl TJ, Hüttenbrink K-B, Hummel T (2002). MR evaluation in patients with isolated anosmia since birth or early childhood. Am J Neuroradiol.

[CR2] Adams DR, Wroblewski KE, Kern DW, Kozloski MJ, Dale W, McClintock MK, Pinto JM (2017). Factors associated with inaccurate self-reporting of olfactory dysfunction in older US adults. Chem Senses.

[CR3] Aschenbrenner K, Hummel C, Teszmer K, Krone F, Ishimaru T, Seo HS, Hummel T (2008). The influence of olfactory loss on dietary behaviors. The Laryngoscope.

[CR4] Bendas J, Hummel T, Croy I (2018). Olfactory function relates to sexual experience in adults. Arch Sex Behav.

[CR5] Bitter T, Gudziol H, Burmeister HP, Mentzel H-J, Guntinas-Lichius O, Gaser C (2010). Anosmia leads to a loss of gray matter in cortical brain areas. Chem Senses.

[CR6] Blomqvist EH, Brämerson A, Stjärne P, Nordin S (2004). Consequences of olfactory loss and adopted coping strategies. Rhinology.

[CR7] Boesveldt S, Postma EM, Boak D, Welge-Luessen A, Schöpf V, Mainland JD, Duffy VB (2017). Anosmia—a clinical review. Chem Senses.

[CR8] Bojanowski V, Hummel T, Croy I (2013). Isolierte congenitale Anosmie-Klinische und alltägliche Aspekte eines Lebens ohne Geruchssinn. Laryngorhinootologie.

[CR9] Borkovec TD, Robinson E, Pruzinsky T, DePree JA (1983). Preliminary exploration of worry: some characteristics and processes. Behav Res Ther.

[CR10] Cavazzana A, Röhrborn A, Garthus-Niegel S, Larsson M, Hummel T, Croy I (2018). Sensory-specific impairment among older people. An investigation using both sensory thresholds and subjective measures across the five senses. PloS one.

[CR11] Cerda-Molina AL, Hernández-López L, Claudio E, Chavira-Ramírez R, Mondragón-Ceballos R (2013) Changes in men’s salivary testosterone and cortisol levels, and in sexual desire after smelling female axillary and vulvar scents. Frontiers in Endocrinology 4 10.3389/fendo.2013.00159 PMC3809382 24194730

[CR12] Costanzo RM, Miwa T (2006) Posttraumatic olfactory loss. In Taste and Smell 63:99–107 Karger Publishers 10.1159/000093753 16733335

[CR13] Croy I, Hummel T (2017). Olfaction as a marker for depression. J Neurol.

[CR14] Croy I, Landis BN, Meusel T, Seo H-S, Krone F, Hummel T (2011). Patient adjustment to reduced olfactory function. Archives of Otolaryngology-Head & Neck Surgery.

[CR15] Croy I, Mohr T, Weidner K, Hummel T, Junge-Hoffmeister J (2019). Mother-child bonding is associated with the maternal perception of the child's body odor. Physiol Behav.

[CR16] Croy I, Negoias S, Novakova L, Landis BN, Hummel T (2012). Learning about the functions of the olfactory system from people without a sense of smell. PLoS ONE.

[CR17] Croy I, Nordin S, Hummel T (2014). Olfactory disorders and quality of life—an updated review. Chem Senses.

[CR18] Croy I, Olgun S, Mueller L, Schmidt A, Muench M, Hummel C, Hummel T (2015). Peripheral adaptive filtering in human olfaction? Three studies on prevalence and effects of olfactory training in specific anosmia in more than 1600 participants. Cortex.

[CR19] Croy I, Viola B, Thomas H (2013). Men without a sense of smell exhibit a strongly reduced number of sexual relationships, women exhibit reduced partnership security–a reanalysis of previously published data. Biol Psychol.

[CR20] De Jong P, Sportel B, De Hullu E, Nauta M (2012). Co-occurrence of social anxiety and depression symptoms in adolescence: differential links with implicit and explicit self-esteem?. Psychol Med.

[CR21] Desiato VM, Levy DA, Byun YJ, Nguyen SA, Soler ZM, Schlosser RJ (2020) The prevalence of olfactory dysfunction in the general population: a systematic review and meta-analysis. Am J Rhinol Allergy 1945892420946254 10.1177/1945892420946254 PMC13080788 32746612

[CR22] Dieterle C, Landgraf R (2006). Folgeerkrankungen und Komplikationen der Adipositas. Der Internist.

[CR23] Doty RL, Shaman P, Applebaum SL, Giberson R, Siksorski L, Rosenberg L (1984). Smell identification ability: changes with age. Science.

[CR24] Doty RL, Shaman P, Kimmelman CP, Dann MS (1984). University of Pennsylvania Smell Identification Test: a rapid quantitative olfactory function test for the clinic. The Laryngoscope.

[CR25] Erskine SE, Philpott CM (2020). An unmet need: Patients with smell and taste disorders. Clin Otolaryngol.

[CR26] Fairburn CG, Doll HA, Welch SL, Hay PJ, Davies BA, O'Connor ME (1998). Risk factors for binge eating disorder: a community-based, case-control study. Arch Gen Psychiatry.

[CR27] Fitzgerald RG, Parkes CM (1998). Blindness and loss of other sensory and cognitive functions. BMJ (Clinical research ed).

[CR28] Frasnelli J, Fark T, Lehmann J, Gerber J, Hummel T (2013). Brain structure is changed in congenital anosmia. Neuroimage.

[CR29] Frasnelli J, Hummel T (2005). Olfactory dysfunction and daily life. European Archives of Oto-Rhino-Laryngology and Head & Neck.

[CR30] Frasnelli J, Schuster B, Hummel T (2007). Subjects with congenital anosmia have larger peripheral but similar central trigeminal responses. Cereb Cortex.

[CR31] Gellrich J, Sparing-Paschke L-M, Thieme T, Schwabe K, Dworschak A, Hummel T, Schriever VA (2019). Normative data for olfactory threshold and odor identification in children and adolescents. Int J Pediatr Otorhinolaryngol.

[CR32] Gottfried JA (2006) Smell: central nervous processing. In Taste and smell 63:44–69 Karger Publishers 10.1159/000093750 16733332

[CR33] Güdücü C, Olcay B, Schäfer L, Aziz M, Schriever V, Özgören M, Hummel T (2019). Separating normosmic and anosmic patients based on entropy evaluation of olfactory event-related potentials. Brain Res.

[CR34] Han P, Hummel T, Raue C, Croy I (2019). Olfactory loss is associated with reduced hippocampal activation in response to emotional pictures. Neuroimage.

[CR35] Havlicek J, Saxton TK, Roberts SC, Jozifkova E, Lhota S, Valentova J, Flegr J (2008). He sees, she smells? Male and female reports of sensory reliance in mate choice and non-mate choice contexts. Personality Individ Differ.

[CR36] Havlíček J, Winternitz J, Roberts SC (2020). Major histocompatibility complex-associated odour preferences and human mate choice: near and far horizons. Philosophical Transactions of the Royal Society B.

[CR37] Hillson R (2014). Taste and smell in diabetes. Pract Diabetes.

[CR38] Hoffman HJ, Rawal S, Li CM, Duffy VB (2016). New chemosensory component in the US National Health and Nutrition Examination Survey (NHANES): first-year results for measured olfactory dysfunction. Rev Endocr Metab Disord.

[CR39] Hofsöe SM, Lehane CM, Wittich W, Hilpert P, Dammeyer J (2019). Interpersonal communication and psychological well-being among couples coping with sensory loss: the mediating role of perceived spouse support. J Soc Pers Relat.

[CR40] Hummel T, Kobal G, Gudziol H, Mackay-Sim A (2007). Normative data for the “Sniffin’Sticks” including tests of odor identification, odor discrimination, and olfactory thresholds: an upgrade based on a group of more than 3,000 subjects. Eur Arch Otorhinolaryngol.

[CR41] Hummel T, Whitcroft K, Andrews P, Altundag A, Cinghi C, Costanzo R, Gupta N (2017) Position paper on olfactory dysfunction. Rhinol Suppl 54(26) 10.4193/Rhino16.248 29528615

[CR42] Kabat-Zinn J (2003). Mindfulness-based interventions in context: past, present, and future. Clin Psychol Sci Pract.

[CR43] Karstensen H, Tommerup N (2012). Isolated and syndromic forms of congenital anosmia. Clin Genet.

[CR44] Keller A, Malaspina D (2013). Hidden consequences of olfactory dysfunction: a patient report series. BMC Ear, Nose and Throat Disorders.

[CR45] Kohli P, Soler ZM, Nguyen SA, Muus JS, Schlosser RJ (2016). The association between olfaction and depression: a systematic review. Chem Senses.

[CR46] Kollndorfer K, Reichert J, Brückler B, Hinterleitner V, Schöpf V (2017). Self-esteem as an important factor in quality of life and depressive symptoms in anosmia: a pilot study. Clin Otolaryngol.

[CR47] Kupers R, Ptito M (2014). Compensatory plasticity and cross-modal reorganization following early visual deprivation. Neurosci Biobehav Rev.

[CR48] Lehane CM (2017) Dyadic Adjustment to Sensory Loss: An investigation of couples’ mental health, support, and coping mechanisms when living with hearing, vision, or dual-sensory loss

[CR49] Lemogne C, Smadja J, Zerdazi E-H, Soudry Y, Robin M, Berthoz S, Bonfils P (2015). Congenital anosmia and emotion recognition: a case-control study. Neuropsychologia.

[CR50] Lim MH, Rodebaugh TL, Zyphur MJ, Gleeson JF (2016). Loneliness over time: the crucial role of social anxiety. J Abnorm Psychol.

[CR51] Lobmaier JS, Fischbacher U, Wirthmüller U, Knoch D (2018). The scent of attractiveness: levels of reproductive hormones explain individual differences in women's body odour. Proceedings of the Royal Society B: Biological Sciences.

[CR52] Lübke KT, Croy I, Hoenen M, Gerber J, Pause BM, Hummel T (2014). Does human body odor represent a significant and rewarding social signal to individuals high in social openness?. PLoS ONE.

[CR53] Lundström JN, Boyle JA, Zatorre RJ, Jones-Gotman M (2009). The neuronal substrates of human olfactory based kin recognition. Hum Brain Mapp.

[CR54] Mahmut MK, Croy I (2019). The role of body odors and olfactory ability in the initiation, maintenance and breakdown of romantic relationships—a review. Physiol Behav.

[CR55] Mattes RD, Cowart BJ, Schiavo MA, Arnold C, Garrison B, Kare MR, Lowry LD (1990). Dietary evaluation of patients with smell and/or taste disorders. Am J Clin Nutr.

[CR56] McBurney DH, Levine JM, Cavanaugh PH (1976). Psychophysical and social ratings of human body odor. Pers Soc Psychol Bull.

[CR57] McLaughlin KA, Borkovec TD, Sibrava NJ (2007). The effects of worry and rumination on affect states and cognitive activity. Behav Ther.

[CR58] Milinski M, Croy I, Hummel T, Boehm T (2013). Major histocompatibility complex peptide ligands as olfactory cues in human body odour assessment. Proceedings of the Royal Society B: Biological Sciences.

[CR59] Miwa T, Furukawa M, Tsukatani T, Costanzo RM, DiNardo LJ, Reiter ER (2001). Impact of olfactory impairment on quality of life and disability. Archives of Otolaryngology-Head & Neck Surgery.

[CR60] Modinos G, Ormel J, Aleman A (2010). Individual differences in dispositional mindfulness and brain activity involved in reappraisal of emotion. Soc Cogn Affect Neurosci.

[CR61] Murr J, Hummel T, Ritschel G, Croy I (2018). Individual Significance of Olfaction: A Comparison Between Normosmic and Dysosmic People. Psychosomatics.

[CR62] Myers A, Rosen JC (1999). Obesity stigmatization and coping: relation to mental health symptoms, body image, and self-esteem. Int J Obes.

[CR63] Nordin S, Brämerson A, Millqvist E, Bende M (2007). Prevalence of parosmia: the Skövde population-based studies. Rhinology.

[CR64] Nordin S, Hedén Blomqvist E, Olsson P, Stjärne P, Ehnhage A, Group NSS (2011). Effects of smell loss on daily life and adopted coping strategies in patients with nasal polyposis with asthma. Acta Otolaryngol.

[CR65] Novakova L, Croy I, Havlicek J, Hummel T (2011) Congenitally anosmic adults report less maternal care than normosmics: a retrospective questionnaire study. Paper presented at the Chemical senses

[CR66] Oleszkiewicz A, Hummel T (2019). Whose nose does not know? Demographical characterization of people unaware of anosmia. Eur Arch Otorhinolaryngol.

[CR67] Oleszkiewicz A, Kunkel F, Larsson M, Hummel T (2020). Consequences of undetected olfactory loss for human chemosensory communication and well-being. Philosophical Transactions of the Royal Society B.

[CR68] Oleszkiewicz A, Park D, Resler K, Draf J, Schulze A, Zang Y, Hummel T (2019). Quality of life in patients with olfactory loss is better predicted by flavor identification than by orthonasal olfactory function. Chem Senses.

[CR69] Olsson MJ, Lundström JN, Kimball BA, Gordon AR, Karshikoff B, Hosseini N, Soop A (2014). The scent of disease: human body odor contains an early chemosensory cue of sickness. Psychol Sci.

[CR70] Olze H, Szczepek AJ, Haupt H, Förster U, Zirke N, Gräbel S, Mazurek B (2011). Cochlear implantation has a positive influence on quality of life, tinnitus, and psychological comorbidity. The Laryngoscope.

[CR71] Oozeer N, Forbes K, Clement A, Kubba H (2011). Management of paediatric olfactory dysfunction: how we do it. Clin Otolaryngol.

[CR72] Orth U, Robins RW (2013). Understanding the link between low self-esteem and depression. Curr Dir Psychol Sci.

[CR73] Parma V, Ohla K, Veldhuizen MG, Niv MY, Kelly CE, Bakke AJ, Dibattista M (2020). More than smell—COVID-19 is associated with severe impairment of smell, taste, and chemesthesis. Chem Senses.

[CR74] Peter MG, Mårtensson G, Postma EM, Nordin LE, Westman E, Boesveldt S, Lundström JN (2020) Morphological changes in secondary, but not primary, sensory cortex in individuals with life-long olfactory sensory deprivation. Neuroimage 117005 10.1016/j.neuroimage.2020.117005 32485304

[CR75] Philpott CM, Boak D (2014). The impact of olfactory disorders in the United Kingdom. Chem Senses.

[CR76] Pinto JM, Wroblewski KE, Kern DW, Schumm LP, McClintock MK (2014). Olfactory dysfunction predicts 5-year mortality in older adults. PLoS ONE.

[CR77] Porter RH (1998). Olfaction and human kin recognition. Genetica.

[CR78] Porter RH, Cernoch JM, McLaughlin FJ (1983). Maternal recognition of neonates through olfactory cues. Physiol Behav.

[CR79] Prazak M, Critelli J, Martin L, Miranda V, Purdum M, Powers C (2012). Mindfulness and its role in physical and psychological health. Applied Psychology: Health and Well-Being.

[CR80] Prehn-Kristensen A, Wiesner C, Bergmann TO, Wolff S, Jansen O, Mehdorn HM, Pause BM (2009). Induction of empathy by the smell of anxiety. PLoS ONE.

[CR81] Rochet M, El-Hage W, Richa S, Kazour F, Atanasova B (2018). Depression, olfaction, and quality of life: a mutual relationship. Brain Sci.

[CR82] Rowan NR, Soler ZM, Storck KA, Othieno F, Ganjaei KG, Smith TL, Schlosser RJ (2019) Impaired eating‐related quality of life in chronic rhinosinusitis. Paper presented at the International forum of allergy & rhinology 10.1002/alr.22242 PMC6397059 30485716

[CR83] Schaal B, Marlier L, Soussignan R (1998). Olfactory function in the human fetus: evidence from selective neonatal responsiveness to the odor of amniotic fluid. Behav Neurosci.

[CR84] Schaal B, Saxton TK, Loos H, Soussignan R, Durand K (2020). Olfaction scaffolds the developing human from neonate to adolescent and beyond. Philosophical Transactions of the Royal Society B.

[CR85] Schablitzky S, Pause BM (2014). Sadness might isolate you in a non-smelling world: olfactory perception and depression. Front Psychol.

[CR86] Schäfer L, Hummel T, Croy I (2019). The design matters: How to detect neural correlates of baby body odors. Front Neurol.

[CR87] Schäfer L, Mehler L, Hähner A, Walliczek U, Hummel T, Croy I (2019). Sexual desire after olfactory loss: quantitative and qualitative reports of patients with smell disorders. Physiol Behav.

[CR88] Schäfer L, Sorokowska A, Sauter J, Schmidt AH, Croy I (2020). Body odours as a chemosignal in the mother–child relationship: new insights based on an human leucocyte antigen-genotyped family cohort. Philosophical Transactions of the Royal Society B.

[CR89] Schäfer L, Sorokowska A, Weidner K, Croy I (2020). Children’s body odors: hints to the development status. Front Psychol.

[CR90] Schriever VA, Agosin E, Altundag A, Avni H, Van HC, Cornejo C, Guarneros M (2018). Development of an international odor identification test for children: the universal sniff test. J Pediatr.

[CR91] Schriever VA, Gellrich J, von der Hagen M, Hummel T (2018). Acquired olfactory dysfunction in children and adolescents: a systematic review of the literature. Chem Senses.

[CR92] Schriever VA, Hummel T (2020) Etiologies of olfactory dysfunction in a pediatric population: based on a retrospective analysis of data from an outpatient clinic. European Archives of Oto-rhino-laryngology: Official Journal of the European Federation of Oto-rhino-laryngological Societies (EUFOS): Affiliated with the German Society for Oto-rhino-laryngology-Head and Neck Surgery 10.1007/s00405-020-06087-4 PMC8249255 32488374

[CR93] Sorokowska A, Pietrowski D, Schäfer L, Kromer J, Schmidt AH, Sauter J, Croy I (2018). Human leukocyte antigen similarity decreases partners’ and strangers’ body odor attractiveness for women not using hormonal contraception. Horm Behav.

[CR94] Star A, Hay P, Quirk F, Mond J (2015). Perceived discrimination and favourable regard toward underweight, normal weight and obese eating disorder sufferers: implications for obesity and eating disorder population health campaigns. BMC obesity.

[CR95] Stevenson RJ (2010). An initial evaluation of the functions of human olfaction. Chem Senses.

[CR96] Temmel AF, Quint C, Schickinger-Fischer B, Klimek L, Stoller E, Hummel T (2002). Characteristics of olfactory disorders in relation to major causes of olfactory loss. Archives of Otolaryngology-Head & Neck Surgery.

[CR97] Van den Bergh O, Stegen K, Van Diest I, Raes C, Stulens P, Eelen P, Nemery B (1999). Acquisition and extinction of somatic symptoms in response to odours: a Pavlovian paradigm relevant to multiple chemical sensitivity. Occup Environ Med.

[CR98] Van Gaal LF, Mertens IL, Christophe E (2006). Mechanisms linking obesity with cardiovascular disease. Nature.

[CR99] Varendi H, Porter R (2001). Breast odour as the only maternal stimulus elicits crawling towards the odour source. Acta Paediatr.

[CR100] Walker A, Pottinger G, Scott A, Hopkins C (2020) Anosmia and loss of smell in the era of covid-19. bmj 370 10.1136/bmj.m2808 32694187

[CR101] Yousem DM, Geckle RJ, Bilker W, McKeown DA, Doty RL (1996). MR evaluation of patients with congenital hyposmia or anosmia. AJR Am J Roentgenol.

[CR102] Zang Y, Han P, Burghardt S, Knaapila A, Schriever V, Hummel T (2019). Influence of olfactory dysfunction on the perception of food. Eur Arch Otorhinolaryngol.

[CR103] Zou L-Q, Vogt O, Schriever VA, Croy I, Schaal B, Hummel T (2020). Decreasing prevalence of specific anosmia to non-steroid odorants from childhood to adolescence. Physiol Behav.

